# The Israeli national population program of genetic carrier screening for reproductive purposes. How should it be continued?

**DOI:** 10.1186/s13584-019-0345-1

**Published:** 2019-12-16

**Authors:** Joël Zlotogora

**Affiliations:** Hadassah Medical School, Faculty of Medicine, Hebrew University of Jerusalem, Jerusalem, Israel

**Keywords:** Carrier screening, Population screening, Jews, Arabs

## Abstract

The Israeli population genetic screening program for reproductive purposes, is a population-specific screening that includes all known, severe diseases and relatively frequent in a specific population (carrier frequency at or above 1:60 and/or disease frequency at or above 1 in 15,000 live births). The carrier screening program is free of charge and offers testing according to disease frequency in the different groups within the population.

The extraordinary technical changes that occurred in the last decade as well as the changes in the type of marriages within the Israeli population necessitate a revision in the basis of the program.

The screening should include instead of only the relatively frequent variants, all the variants that were reported among patients causing a severe disease for which the natural history is well known without regard of their frequency. The population-specific screening that determine which variants are included according to the origin of the couple should be abandoned for a general screening including either all the Jewish population or all the Israeli Arab population.

The initiation in 1971 of a screening program to prevent Tay Sachs disease among Ashkenazi Jews in the United States led to the establishment in 1978 of a national carrier screening program in Israel under the aegis of the Ministry of Health [1, 2]. Carrier screening for the prevention of β-thalassemia was subsequently added to the national program for the Arab population as well as for the Jewish communities in which the disease is relatively frequent [3]. The progressive ability to perform a molecular diagnosis of many genetic diseases relatively frequent in the Israeli population has made many disorders candidates for screening. These screening tests were offered privately and later were partially covered by non-mandatory supplementary health insurance held by three-fourths of the population. In 2004, the Association of Israeli Medical Geneticists recommended including in the national carrier-screening program for reproductive purposes all the severe genetic diseases with a carrier frequency at or above 1:60 or with a disease incidence at or above 1 in 15,000 live births in a specific population in which there is a test available that will detect more than 90% of the carriers. In addition, the Association of Israeli Medical Geneticists suggested to consider the possibility to screen for severe diseases that are less frequent [2].

Due to budgetary limitations, the Ministry of Health gradually sponsored the expansion of the Israeli program. In 2002 the first step was to add to the Tay Sachs and thalassemia carrier program, screening severe genetic diseases with a frequency at or above 1:1000 live births in a specific population which was de facto aiming at the Arab communities [4]. In 2008, the second step was to add screening for cystic fibrosis in almost all the Israeli population and familial dysautonomia among Ashkenazi Jews. Since January 2013, the Israeli population genetic screening program for reproductive purposes is a population-specific screening that includes all known, severe diseases that are relatively frequent in a specific population (carrier frequency at or above 1:60 and/or disease frequency at or above 1 in 15,000 live births) according to the recommendations of the Association of Israeli Medical Geneticists and it is updated every year according to new data [5] (additional file 1). Individuals may choose to have the tests; either through the general program usually as sequential screening, the woman being first examined and her partner only if necessary or through the “Dor Yeshorim” couple screening program. The “Dor Yeshorim” program is aimed to the ultra-orthodox Jewish community in which the marriages are arranged and the “genetic compatibility” of the prospective spouses is determined [6].

The tests included in the national program are free of charge while genetic tests for diseases that are less frequent but recommended for consideration by the Association of Israeli Medical Geneticists are offered in the context of supplementary insurances. The possibility to perform other tests is offered as a private choice in the context of genetic counselling. Couples at risk for an affected child receive at no cost genetic counselling in which all the reproductive options are discussed such as changing the marriage plan, prenatal diagnosis, preimplantation genetic diagnosis. Another possibility is not to intervene with the option of the examination of the newborn and early treatment of the child if affected.

The extraordinary technical changes that occurred in the last decade in particular the introduction of next generation sequencing allowing much more rapid and cheaper sequencing [7], as well as the changes in the type of marriages within the Israeli population necessitate a revision of of the program. This commentary discuss ways the program should evolve.

## Which diseases/variants should be included in the screening?

### Disease frequency

In the recommendation of the Association of Israeli Medical Geneticists of screening for diseases with frequency at or above 1:15,000 (carrier frequency at or above 1:60 for autosomal recessive diseases) in a specific population was chosen mainly the consequence of the cost as well as the clinical utility of the tests. With the changes and advances in technologies, the marginal cost of adding variants to the existing panel is relatively small. In parallel, it is possible to reduce either the false positive rates using alternative techniques to confirm the carrier status or the uncertainty of the results by the inclusion only of variants of known significance.

### Variants based screening or screening by gene sequencing

Variants that have been reported among patients affected with autosomal recessive diseases in the Israeli population have been collected for more than two decades and are included in the Israeli National Genetic Database (INGD) (http://INGD.huji.ac.il) [8]. In addition, results of whole exome/whole genome sequencing of 5685 Ashkenazi Jews either affected with inflammatory bowel disease or random non affected individuals are available in gnomAD (https://ibd.broadinstitute.org) including variant frequencies in this population [9]. Comparing these two sources among Ashkenazi Jews, shows that some variants that were considered at the time of their publication as pathogenic since they were detected in affected patients, are found frequently in homozygosity among unaffected individuals and are probably benign. On the other hand, other variants that are pathogenic and according to their allele frequency in gnomAD should have been diagnosed in Ashkenazi Jewish patients are not present in INGD. An example is the frameshift variant c.428delG in *KIAA0586* that was reported in several patients with Joubert syndrome 23 [10]. In gnomAD, this variant was reported in all populations including two non-Ashkenazi homozygotes. Among Ashkenazi Jews the c.428delG variant is particularly frequent (allele frequency = 0.008068) however, Joubert syndrome 23 has not yet been reported. In almost all patients with Joubert syndrome 23 in whom the variant c.428delG was detected it was found in compound heterozygosity with another variant. It seems that in homozygotes for the variant c.428delG clinical symptoms are present only when another event exist, such as a variant in another ciliopathy gene [10].

### Variants for which the natural history of the disease is known

In many genes some of the variants are responsible for a severe disease while other for a much milder one. As an example among the variants reported among patients with Gaucher disease the variant c.1226A > G (p. Asn409Ser) in *GBA* that is frequent among Ashkenazi Jews always leads to a non neuronopathic type of the disease and most homozygotes are asymptomatic [11]. On the other hand, several other variants are associated with severe forms of the disease. The decision to include severe diseases in the screening program should also take in account the variants screened.

Another type of problem is that the natural history of the disease caused by a specific variant is not known. An example is the pathogenic variant c.964-1G > C in *DHCR7* responsible for Smith-Lemli-Opitz syndrome in many populations [12]. A very high frequency of the variant among Ashkenazi Jews screened using universal screening [13] led to the Association of Israeli Medical Geneticists recommendation to include Smith-Lemli-Opitz syndrome carrier screening among Ashkenazi Jews and its inclusion in the Israeli national program in 2017 “(https://www.health.gov.il/Subjects/Genetics/Documents/seker_sal2017.pdf)”. While the variant is reported in gnomAD with an allele frequency of 0.01170 among Ashkenazi Jews (113 alleles, no homozygotes) [9], the syndrome is not known to be frequent in this population. To the best of our knowledge only one patient homozygote for the variant c.964-1G > C was diagnosed in the recent years among Ashkenazi Jews in Israel (Daum et al., submitted). It is probable, as it has been suggested, that most of the fetuses that are homozygotes are aborted in an early stage of the pregnancy [12]. However, even if theoretical assumptions may be made, there are not enough available data in the literature to determinate the definite risk of the couples at risk to have an affected child. The genetic counselling of couples discovered to be at risk for an affected child through a screening test is complex specifically in regards to choosing the best way of prevention. The possibility to choose between preimplantation or prenatal diagnosis either early with chorion villi sampling or late with amniocentesis must be based on the knowledge of the risk for a living affected child. The decision to include screening for Smith-Lemli-Opitz syndrome in a population-screening program should have been made only after the completion of a study aimed to determine the risk of couples at risk.

*The Israeli program should include only variants for which the natural history of the disease is known and is severe. The expansion of the program should be for less frequent severe diseases in particular those that the Association of Israeli Medical Geneticists recommended for consideration (carrier frequency at or above 1:100 for autosomal recessive diseases). An even better option would be to include all the pathogenic variants, as characterized via genomic analysis of affected patients that have been reported to be affected with severe autosomal recessive diseases in the Israeli population*

## Population-specific screening program or universal screening

While marriages are mostly within the religious groups, in each group inter-community marriages are increasingly frequent. Among Jews, the Ashkenazi represent the largest community and there are still marriages in which all 4 grandparents of the spouses are Ashkenazi Jews, while in the other communities among spouses born in Israel, couples in which the 4 grandparents have the same origin are rare. Even more, many individuals do not know the exact origin of their family. In addition, some of the variants that have been reported among patients affected with the disease in the Jewish population are found in more than one community in lower frequency [8]. Among Arabs, marriages between spouses from different localities are becoming more frequent and many variants frequent in one locality have been diagnosed in patients in other localities.

The universal screening approach may be problematic since a variant pathogenic in one population may be benign in the other. The variant c.579G > A (p.Trp193Ter) in *NCF1* is a relatively frequent founder variant among Kavkazi Jewish patients affected with chronic granulomatous disease [14]. The variant is also frequent among Ashkenazi Jews although Ashkenazi chronic granulomatous disease patients with this variant have not been described [8, 9]. Carrier screening based on a uniform expanded pan Israeli preconception screening panel of an Ashkenazi couple while the woman was pregnant revealed that both spouses were heterozygotes the variant c.579G > A, and prenatal diagnosis was performed [15]. The fetus inherited both carrier alleles but was not affected; having substantial oxidase activity revealed by the analysis of fetal leucocytes obtained by cordocentesis. While in the 7q11.23 region the typical arrangement includes one *NCF1* gene and one pseudogene, each of the parents carried one allele with the pseudogene and two complete copies of the *NCF1* gene, one of these containing the c.579G > A variant. The fetus received one abnormal allele from each parents and therefore had 4 copies of the *NCF1* gene of which two with the variants and two functional genes. Even though in both Ashkenazi and Kavkazi Jews the carrier rate of the variant c.579G > A in *NCF1* is relatively high, the variant is probably pathogenic only in the Kavkazi Jews due to differences in the structure of the region of the gene.

*Since inter religious marriages are rare while inter-communities marriages are frequent, the Israeli program should include, based on cost effectiveness criteria either two distinct panels one for the Jewish population and the other for Arabs or a single panel for the entire population.*

## Individual or couple screening

In the Israeli program, carrier screening is almost always sequential, performed first in one of the spouses. The woman is usually the individual examined since the fragile X screening is done only in women. If the first individual is found to be a carrier for a specific disease, her partner is also tested for this disease. In the premarital screening offered in the ultra-orthodox Jewish community (“Dor Yeshorim” program), both potential spouses are tested and individual results are not given (couple screening). The results are given as a couple and the marriage will be avoided if both members of the prospective couple are carriers of the same autosomal recessive gene.

If both partners of a couple are examined, a major concern may be additional costs since in sequential screening the partner is examined only when the first individual examined is a carrier. However, since in couple screening the individual results are not given, while DNA is taken from both partners in a first stage only one individual is examined and the other is examined only if found to be a carrier.

Since the purpose of carrier screening for reproductive purposes is to detect couples at risk for an affected child, the individual knowledge about being a carrier is incidental [16]. This knowledge is often considered important because it allows relatives to be informed about the existence of the variant in the family and then to perform cascade screening. However, when a population screening program exists, cascade screening is less relevant since carrier screening is recommended to all, whether or not a variant is known to exist among the relatives. On the other hand, sequential screening may worry the individual found to be a carrier. This type of event is becoming more frequent since the probability to discover that the individual screened is a carrier rise as a result of the increased number of tests performed [13]. The knowledge about the carrier state often focuses the attention on the disease for which previous knowledge of the individual was minimal, learning of its severe complications. This may be stressful in particular in a pregnant woman until the partner is examined. Anxiety may remain even if the partner is not found to be a carrier since a residual risk for the fetus to be affected with the disease remains. Another problem is that, in some cases, such as Gaucher disease the carrier status is in fact a presymptomatic knowledge about the risk for late onset symptoms. Variants in the glucocerebrosidase gene are risk factors for Parkinson’s disease and it has been suggested that variants in genes related to other lysosomal storage disorders are also associated with disease susceptibility [17]. In order to fulfill its goal, carrier screening for reproductive purposes need to report only about the existence of the risk for an affected child.

*Carrier screening has been available for decades in Israel and for all these years the results are given to the individuals tested, therefore, this approach will be difficult to change. However, the option of couple screening and its advantages should be explained and made available before performing the tests. Testing for carrier status should be available to those interested as well as presymptomatic knowledge for late onset disorders.*

## Conclusions

An important criterion, according to the guidelines for population screening first delineated by Wilson and Jungner for the World Health Organization and later applied to population genetic screening [18] is the possibility of an effective intervention. For a screening program aimed to reproductive purposes, the intervention is to give the couple at risk autonomous, informed choices including prevention. In a population screening program offered by the state such as the one in place in Israel the severity of the diseases/variants screened should warrant the choice of prevention. The changes to the Israeli program suggested include screening for variants causing severe diseases that have been reported in the population in which the program is offered without taking in account their frequency. In order to reduce the anxiety that may be secondary to such type of screening the possibility of couple screening reporting only for results increasing the risk of the couple should be the recommended option. Since new variants causing severe diseases in the Israeli population are constantly reported, a panel such as the one proposed should be updated at least once a year. While new couples will be offered the updated panel, couples that have been screened in the past should be informed about the possibility to complete the screening when only for relatively frequent variants the screening is funded by the public health program.

Additional and different possibilities for genetic screening should also be explained to all the individuals referred to the Israeli population genetic screening program for reproductive purposes as a potential choice in particular screening for less severe diseases and/or presymptomatic testing. These options should be available in a different setting.

There are currently many options to perform expanded carrier screening for reproductive purposes available privately. The potential to use one of these options for population screening is currently examined in several pilot studies (personal communication). At this stage the Israeli program should evolve to include the changes proposed in this comment and not only by the yearly addition of tests such as it has been done in the last years. The final decision on how the Israeli national population program of genetic carrier screening for reproductive purposes should be continued must also take account of the added costs of the proposal. Economic aspects were not discussed but it should be reminded that compliance for genetic screening is lower for tests that are not in the basket of services, even if they are recommended, being perceived as less important [19].

## Additional file

**Additional file 1.** The Israeli population genetic screening program for reproductive purposes. Genetic tests recommended and available free of charge in 2019 according to the origin of the spouses.

## Data Availability

Data sharing is not applicable to this article as no datasets were generated or analyzed during the current study.

## Abbreviations

gnomAD: Genome Aggregation Database
INGD: Israeli National Genetic Database
